# Chondroblastoma of proximal tibia diagnosed by arthroscopy-guided biopsy: a case report

**DOI:** 10.11604/pamj.2023.45.101.40169

**Published:** 2023-06-22

**Authors:** Viresh Kannure, Swapnil Date, Deeksha Rana, Aman Agrawal, Prashant Bhalusani

**Affiliations:** 1 Department of Orthopaedics, Jawaharlal Nehru Medical College, Sawangi (Meghe), Wardha, Maharashtra, India,; 2 Department of Pathology, Jawaharlal Nehru Medical College, Sawangi (Meghe), Wardha, Maharashtra, India

**Keywords:** Intrasynovial chondroblastoma, chondroid cells, diagnostic arthroscopy, chicken wire calcifications, case report

## Abstract

Chondroblastoma considered a rare form of osseous neoplasm contributes less than 1% of all bone tumours. It is typically found in young patients with chief complaints of moderate pain with joint stiffness. It develops as a lytic lesion in the epiphysis of long bones which might spread to the metaphysis. We report a case of an 18-year-old patient who presented with progressive right knee pain which aggravated with movements. Investigations included X-ray, magnetic resonance imaging (MRI), and biochemical assessment. A focal, well-defined, lesion in the upper end of the tibia with surrounding marrow oedema was observed and diagnostic arthroscopy was taken for management. Histopathology of specimen observed chondroblasts proliferation with areas of mature cartilage, and giant cells confirming intrasynovial chondroblastoma. Usually, surgery is the treatment of choice; however, possibilities of the secondary bone cyst, haemosiderin deposition on the joint, etc., make treatment approaches uncertain. Diagnostic arthroscopy is a rare but essential modality in such cases due to better visuals, complete tumour excision, and combination with adjuvant therapies. Chondroblastoma, if untreated, proves detrimental, hence, a thorough evaluation is critical for overall better outcomes.

## Introduction

Chondroblastoma is a benign tumour composed of chondroblasts which are known to be chondroid producing neoplasms. Approximately, less than 1% of all bone tumours are chondroblastoma, and considered as rare form of osseous neoplasm. Typically, in young individuals, it develops in the apophysis or epiphysis of long bones and may spread into the metaphysis [1]. The term ‘chondroblastoma’ was coined by Jaffe *et al*. 1942 which means unorganized and poorly formed bone matrix and collection of immature chondroid cells [2]. Chondroblastoma mostly occurs in the long bones and proximal humerus is most common site of occurrence followed by distal femur and proximal femur [3]. Studies have reported the site of origin is the secondary ossification centre of epiphyseal plate, male to female ratio approximately 3: 2 and recurrence in about 15% cases [4]. Intrasynovial chondroblastoma, though benign, needs quick management approach because the tumour may cause unusual fractures or distant metastasis by spread to vital organs most commonly lungs. Some studies have shown cases with extensive metastatic spread and death [5]. On the basis of prognosis, recurrence rate is low and around 80-90% recurrent cases are simply treated through curettage with bone grafting. A study showed recurrent cases occurring mainly after two years of surgery and mainly in cases of temporal bone site [6]. We present a rare case report of an 18-year-old female patient with initial presentation of progressive right knee pain which was constant and aggravated with movements, underwent diagnostic arthroscopy-guided biopsy, with pathologically proven intrasynovial chondroblastoma and explain our study on the basis of clinical findings, histopathological report and radiodiagnosis.

## Patient and observation

**Patient information:** an 18-year-old female presented with chief complaints of pain over right knee for four years, due to which she was unable to walk. The pain was gradual in onset and progressive in nature. Severity had increased in the last month and was hindering her daily activities. The pain was constant throughout the day, aggravated by movements, and reduced by medications and rest. There was no diurnal variation. There was no history of heavy weight lifting or trauma. The bladder and bowel involvement were present. Patient had no significant family history.

**Clinical findings:** spinal curvature was normal, and the local temperature was not raised. All the deep tendon reflexes were elicited. Higher functions and cranial nerves were intact. On examination of right knee, overlying skin was normal, no scars, sinuses or swelling was seen. Flexion deformity was present and diffuse tenderness around proximal tibia was palpable. The range of motion of knee was 5 degrees to 110 degrees and further painful. Ligaments were intact and special test for meniscus was negative. Active ankle and toe movements were present. Also, dorsalis pedis artery and posterior tibial artery were palpable.

**Figure 1:**
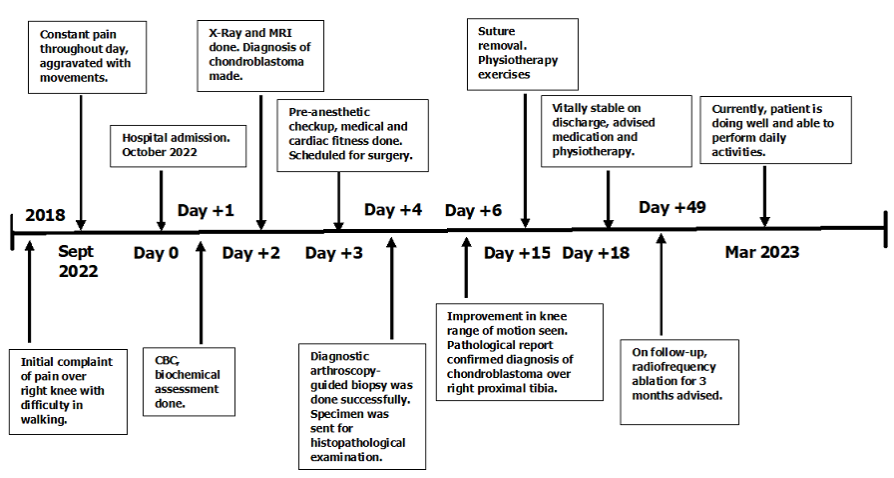
timeline of entire course of disease

**Timeline:** patient was admitted in orthopaedic ward. On admission, patient had no history of hypertension or diabetes mellitus. Routine pre-operative investigations such as complete blood count (CBC), biochemical assessment and pre-anaesthetic check-up was done which were within normal limits (Figure 1).

**Figure 2:**
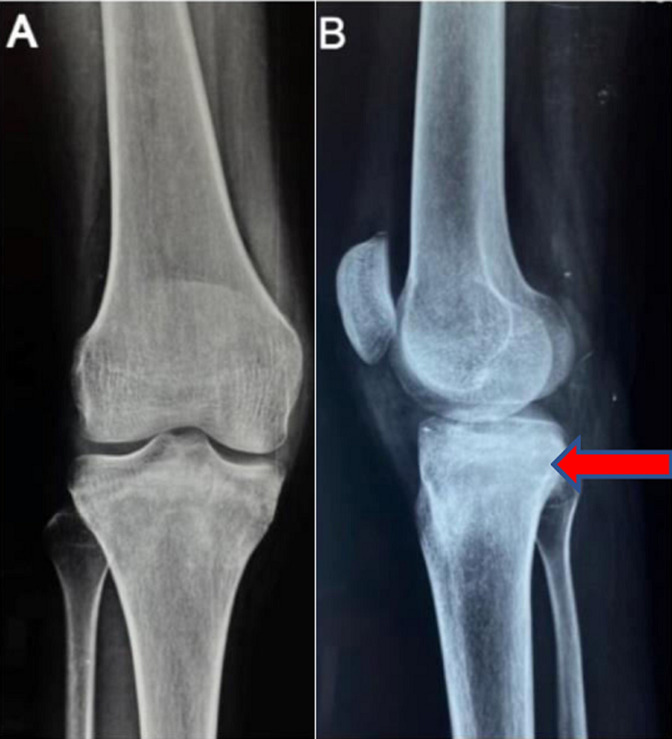
X-ray of right knee joint: A) the anterior-posterior view showing increased bone mineralisation and swelling of soft tissue over proximal tibial region; B) the lateral view showing well defined circular, lytic lesion over posterior aspect of proximal tibia

**Figure 3:**
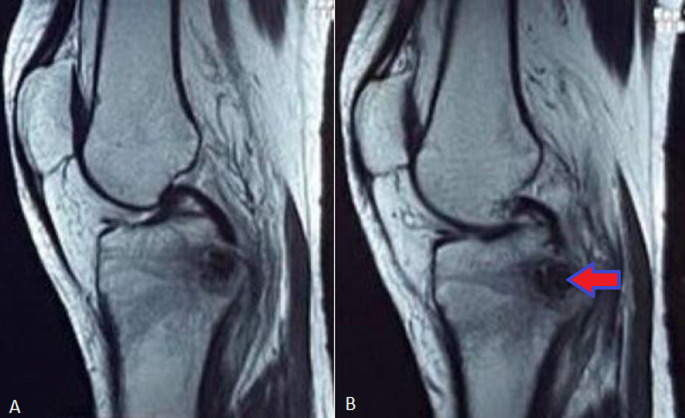
magnetic resonance imaging of right knee joint: A) presence of significant joint effusion in patella-femoral and tibia-femoral joint space; B) a focal, well defined altered signal intensity seen in the upper end of tibia with surrounded marrow oedema

**Diagnostic assessment:** the patient underwent X-ray of right knee in antero-posterior and lateral view which revealed increased bone mineralization in the proximal tibia and soft tissue swelling (Figure 2). The magnetic resonance imaging (MRI) of right knee, revealed a focal, well defined, signal intensity alterations in the upper end of the tibia with surrounding marrow oedema. Lesion is seen in the posterior aspect and measures approximately 14x12.1 CMS (Figure 3).

**Diagnosis:** thorough physical examination and radiological imaging, a clinical diagnosis of intrasynovial chondroblastoma over right proximal tibia was made and patient was posted for diagnostic arthroscopy.

**Figure 4:**
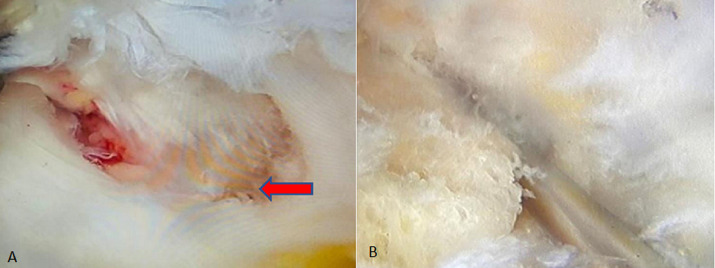
diagnostic arthroscopy of right proximal tibia: A) bony irregularities were seen below the attachment of posterior cruciate ligament; B) bone biopsy needle was inserted through the bony irregularity

**Figure 5:**
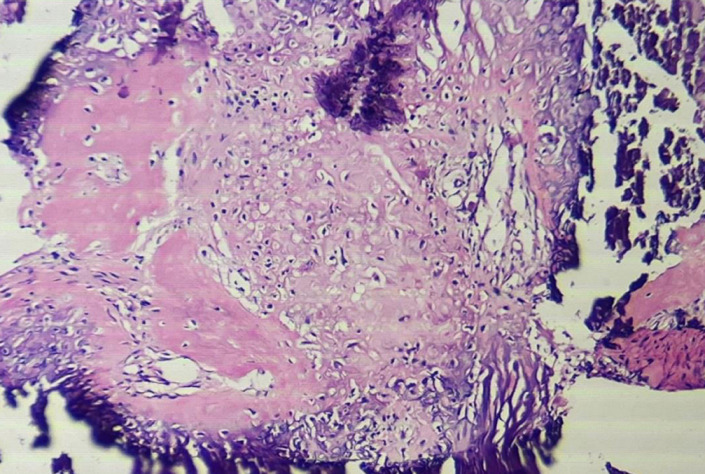
under 40X microscopy, histopathological section of specimen showing chicken wire calcification and chondroid matrix

**Figure 6:**
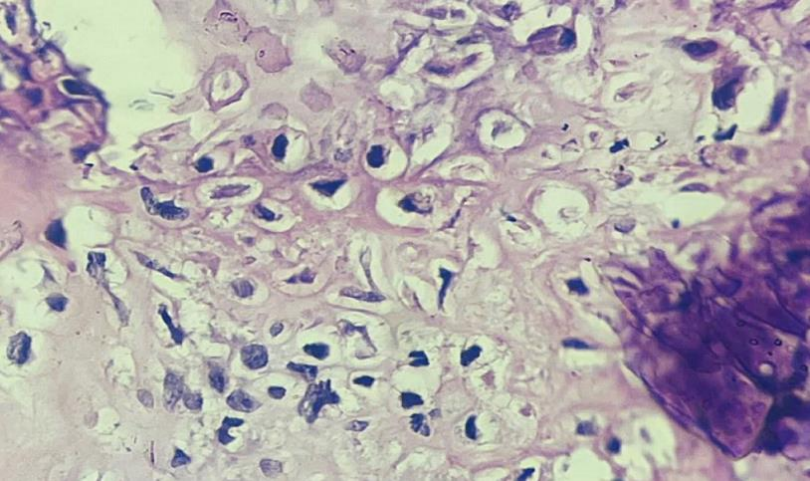
under 400X microscopy, histopathological section of specimen showing sheets of chondroblast cells

**Therapeutic interventions:** standard anteromedial, anterolateral and posteromedial ports were made and diagnostic arthroscopy of right knee with biopsy of the lesion was done under spinal anaesthesia. Suprapatellar pouch, medial and lateral gutter were found to be normal. Anterior cruciate ligament, posterior cruciate ligament and both menisci were found to be normal. Bone irregularities were seen just below the attachment of posterior cruciate ligament. Site was confirmed with image intensified and biopsy was taken with bone biopsy needle under direct visualization and C-arm control and the specimen was sent for histopathological examination to confirm the diagnosis (Figure 4). Long knee brace application was done. Further, antibiotic coverage and pain relief were managed. Grossly, the specimen measured approximately 1x0.5x0.2 cm, was whitish, irregular elongated tissue. Microscopic examinations show tumour tissue arranged in sheets, individual cells are round to polyhedral with hyperchromatic nuclei and abundant eosinophilic cytoplasm. Background shows chondroid matrix with pericellular (chicken-wire) calcification (Figure 5, Figure 6).

**Follow-up and outcome of interventions:** the improvement in the range of motion of knee from 90 degrees to 130 degrees was noted. On day 11 of surgery, no discharge from suture line, no signs of any infection were noted and sutures were removed. The patient was advised post-operative physiotherapy. The patient was vitally stable on discharge and was advised to continue oral medications, ankle pumps, static and dynamic hamstrings and quadriceps exercises, knee range of motion exercises, and full weight bearing mobilization with the help of walker. On one month of follow-up, the patient was advised radiofrequency ablation for a period of three months.

**Patient perspective:** currently, she is doing well and able to perform her daily activities. The patient and her relatives expressed gratitude for the care and treatment received and expressed optimism about her progress.

**Informed consent:**the patient gave written informed consent for publication. The study involving radiological and histopathological images were de-identified to protect patient's privacy and maintain confidentiality.

## Discussion

Diagnostic arthroscopy is a rare, however, becoming quite an essential diagnostic and treatment modality in the cases of chondroblastoma as only surgical excision of such tumour becomes difficult due to anatomical proximity between epiphysis and articular cartilage [7]. The case report presented is singular and rare because of its unusual treatment approach, i.e., diagnostic arthroscopy with biopsy of lesion followed by post-operative radiofrequency ablation. To our knowledge, we found similar instance in only one report published by Kellish *et al*. 2021, who reported case of 15-year-old male diagnosed with chondroblastoma within medial femoral condyle and underwent arthroscopic assisted tumour excision; case of a 14 -year-old male diagnosed with chondroblastoma of medial trochlea and underwent arthroscopy and case of a 15-year-old female diagnosed with chondroblastoma in lateral trochlea through needle biopsy and also underwent arthroscopy [7].

It is reported that chondroblastoma is commonly seen in males, during adolescence with mean age of 27 years [8]. In case discussed here, we report a female patient diagnosed at 18 years of age. Chondroblastoma most commonly occurs in sites such as epiphysis of long bones, flat bones such as ilium and acetabulum, although, sites like patella, talus and calcaneum have also been reported [8]. The differential includes osteomyelitis, chondrosarcoma, chondromyxoid fibroma (CMF), intraosseous ganglion etc., with giant cell tumour being most common. Giant cell tumour can be differentiated from chondroblastoma, as the former lacks sclerotic rim of lesion and seen in skeletally mature patients [9].

Usually in cases of intrasynovial chondroblastoma, curettage with or without bone graft is performed. In this case report, the patient underwent diagnostic arthroscopy [8]. The characteristic advantages of diagnostic arthroscopy include better visual within the lesion, complete excision of tumour, post-operative cartilage integrity assessment and can be combined with adjuvant therapies, making it the treatment of choice [7]. In the case discussed, diagnostic arthroscopy along with biopsy of the lesion was performed as primary approach which is contradictory to the study by Shimizu *et al*. 2018, who reported use of arthroscopy in patients limited to tumours of size approximately 1 cm, lack epiphyseal involvement and non-chondroblastoma cases [10]. However, the procedure has its own limitations, most commonly, continuous bleeding during an intralesional excision case such as intraosseous haemangioma, aneurysmal bone cyst etc., making visualization difficult throughout the surgery [7]. Other treatment modalities include cauterization of curetted lesion with chemical such as phenol, radiotherapy, marginal resection, etc., though this field needs more studies [8].

## Conclusion

Intrasynovial chondroblastoma is the rare neoplastic condition with uncertain risk factors, and pathogenesis unknown resulting in poor prognosis and short survival outcome in some cases. The mainstay for treatment is the traditional surgical curettage and bone grafting, hence, the approach of diagnostic arthroscopy makes our case exceptional. The technique of diagnostic arthroscopy-guided biopsy is quite becoming the management approach because, benign tumours in the tibial region, primarily in posterior of tibial articular area can be treated. Also, direct visualisation, cartilage monitoring during the entire course of operation, possible combined adjuvant therapies etc., are some of its characteristic advantages, however, more research studies are needed to manage cartilage damage possibility. Radiodiagnosis and histopathological confirmation of tumour serves as the prerequisites for radiofrequency ablation during the follow-ups as post-operative radiofrequency is possible for lesions more than 5 cm in size which favoured in our case. Due to scarcity of data regarding diagnostic arthroscopy, this study aids in reviewing outcome of such cases which would help to provide the importance of modern treatment modalities resulting in good patient prognosis and overall long-term survival.
